# Protecting Skeletal Muscle with Protein and Amino Acid during Periods of Disuse

**DOI:** 10.3390/nu8070404

**Published:** 2016-07-01

**Authors:** Elfego Galvan, Emily Arentson-Lantz, Séverine Lamon, Douglas Paddon-Jones

**Affiliations:** 1 Center for Rehabilitation and Physical Activity and Nutrition (CeRPAN), University of Texas Medical Branch, Galveston, TX 77555, USA; elgalvan@utmb.edu (E.G.); ejlantz@utmb.edu (E.A.-L.); 2 Department of Nutrition and Metabolism, University of Texas Medical Branch, Galveston, TX 77555, USA; 3 Institute for Physical Activity and Nutrition (IPAN), School of Exercise and Nutrition Sciences, Deakin University, Geelong 3125, Australia; severine.lamon@deakin.edu.au

**Keywords:** leucine, bed rest, muscle health, inactivity, muscle protein synthesis, diet

## Abstract

Habitual sedentary behavior increases risk of chronic disease, hospitalization and poor quality of life. Short-term bed rest or disuse accelerates the loss of muscle mass, function, and glucose tolerance. Optimizing nutritional practices and protein intake may reduce the consequences of disuse by preserving metabolic homeostasis and muscle mass and function. Most modes of physical inactivity have the potential to negatively impact the health of older adults more than their younger counterparts. Mechanistically, mammalian target of rapamycin complex 1 (mTORC1) signaling and muscle protein synthesis are negatively affected by disuse. This contributes to reduced muscle quality and is accompanied by impaired glucose regulation. Simply encouraging increased protein and/or energy consumption is a well-intentioned, but often impractical strategy to protect muscle health. Emerging evidence suggests that leucine supplemented meals may partially and temporarily protect skeletal muscle during disuse by preserving anabolism and mitigating reductions in mass, function and metabolic homeostasis.

## 1. Introduction

Without a concerted effort to remain physically active and follow sound dietary practices, muscle mass and strength begins to decline by approximately 0.8%/year after the age of 40 years. By the time an individual reaches 65 years, the rate of loss may have increased to 1.5%/year [1,2,3,4]. Short-term bed rest or periods of disuse can accelerate the loss of muscle mass, function, and impair glucose tolerance (Table 1). Inactivity is a common feature of many clinical environments such as hospitals and extended care facilities. Modifying habitual diet and physical activity practices are conceptually easy but practically challenging strategies to counter the effects of disuse. Even in healthy research volunteers, muscle health (i.e., mass, function, metabolism) is compromised during periods of disuse. Inattention to either diet or physical activity or the presence of additional catabolic stressors such as insulin resistance, injury or illness, particularly in older adults, further accelerates inactivity-induced functional and metabolic decline [5,6].

Various disuse models can be used to mimic physical inactivity [7,8,9]. In bed rest models, older adults lose more muscle mass and functional capacity than younger populations [10,11]. Even short periods of disuse can negatively impact muscle health. In older adults, as little as five days of bed rest substantially impact leg extension peak torque (−16%) and lean leg mass (−0.5 kg) [10]. Similar rapid changes have also been reported in healthy middle-aged adults who lost ~1 kg of lean leg mass after seven days of bed rest [7]. These data remind us that advanced aged or the overt presence of sarcopenia is not necessarily a prerequisite for rapid inactivity induced muscle/function loss.

By continuing to refine research models of physical inactivity, we hope to better understand and target the mechanisms regulating muscle and metabolic dysfunction [12]. While disuse models provide a platform for a variety of descriptive and intervention studies, we suggest that dietary approaches to protect muscle health should be viewed as a fundamental prerequisite or companion to exercise and/or pharmacological support. For example, while exercise can clearly counter the negative effects of disuse [6], it may not always be feasible, or of sufficient quantity, duration or intensity to be beneficial in clinical or compromised populations. In contrast, there are few if any circumstances where nutrition cannot be optimized as part of a coordinated strategy to maintain health.

## 2. Protecting Muscle Health through Dietary Manipulation

At the most basic level, dietary strategies to preserve or improve muscle health focus on optimizing energy and protein intake. Beyond the risk of exceeding daily energy requirements and gaining body fat, chronic consumption of as much as 2 g protein/kg/day is generally regarded as safe [13].

While extremely high protein consumption (e.g., >3 g of protein/kg/day) may be tolerated by some individuals [14,15], it may be challenging to meet all macro- and micro-nutrient needs while maintaining energy balance. For the vast majority of healthy, ambulatory adults, consuming more than approximately 1.5 g protein/kg/day will likely provide limited additional benefit [12]. In addition, variation in age, body size, body composition goals, health status and physical activity levels complicate the prescription of specific “*optimal protein consumption goals*” for individuals in a broad, heterogeneous community.

## 3. Protein and Targeted Amino Acid Interventions during Inactivity

Current protein recommendations do not discriminate between young and older adults. The Institute of Medicine (IOM) states that 0.66 g protein/kg/day (Estimated Average Requirement, EAR) meets the needs of about 50% of healthy adults, 19 years and older. The Recommended Dietary Allowance (RDA: 0.80 g protein/kg/day) is an estimate of the minimum daily average dietary protein required to meet the needs of over 97% of the healthy adult population [16]. While the EAR and RDA are often interpreted, erroneously, as desirable average and upper-limit targets [17], dietary protein intake at, or marginally above, the RDA is not sufficient to protect muscle mass and function during inactivity, especially among older adults [7,10,18,19,20,21,22,23].

An acknowledged limitation of the IOM’s protein recommendations is that they target healthy adults and not clinical populations. Consequently, if we accept that disuse models represent a catabolic and metabolically harmful state; direct application of standard protein recommendations may not be warranted. Meeting the minimum amount of protein to maintain nutritional adequacy is clearly conceptually different than consuming protein as a means to counter disuse-related changes in metabolism and muscle health [17,24,25].

**Figure 1:**
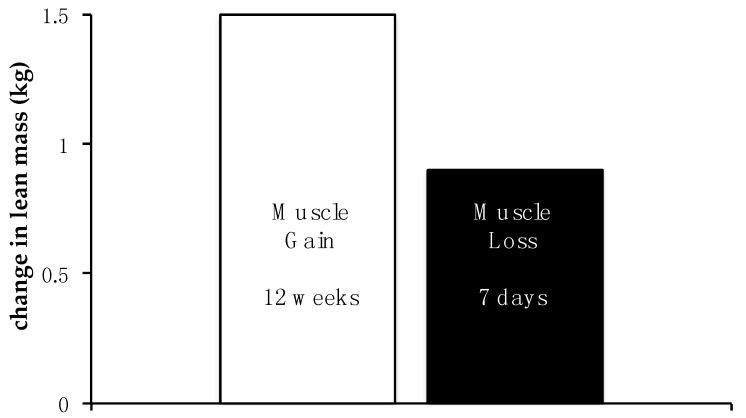
Gains in muscle mass and function due to exercise require regular training over an extended period of time. Twelve weeks of resistance exercise training result in a modest gain (~1.5 kg) in muscle mass in older adults [1]. However, loss of muscle health due to disuse occurs over a very short period of time; seven days of bed rest is sufficient to induce ~1 kg loss leg lean mass alone. Given the effort necessary to maintain muscle health, especially during aging, strategies that protect muscle during disuse are critical.

Most experts agree that meeting nutrient and metabolic needs via a “food-first” approach is desirable (Figure 1). However, in some circumstances, leucine supplementation may be an attractive option due to its ability to stimulate translation initiation and muscle protein synthesis following low-to-moderate protein-containing meals [26,27,28]. Supplemental leucine may also reduce muscle protein breakdown, although this outcome is perhaps less likely to be demonstrably elevated in relatively healthy populations, such as bed rest study participants [29].

Leucine continues to be investigated as an intervention to protect muscle health during inactivity [7]. In healthy middle-aged adults, 3–4 g leucine/meal (0.06 g leucine/kg body mass/meal) partially protected leg lean mass during the first week of a 14-day bed rest study [7]. The protective effect appears to be largely due to a blunted, 10% ± 10% reduction (i.e., partial preservation) in muscle protein synthesis, compared to a much larger, 30% ± 9% reduction in the control group. Notably, the leucine-mediated protection of lean mass was accompanied by the partial preservation of muscular strength, endurance, and quality (peak torque/kg leg lean mass). These data lead us to speculate that leucine may also have the potential to improve outcomes in a clinical setting, although confirmatory clinical trials are certainly needed. In particular, it is questionable if the beneficial anabolic effects of leucine can be maintained for a prolonged period. For example, during the final seven-day of our two-week bed rest protocol, the loss of lean leg mass in leucine supplemented and control subjects was similar [7].

β-Hydroxy β-Methylbutyrate (HMB), a leucine metabolite, has also been used to protect muscle mass during bed rest. HMB supplementation (3 g HMB/day) during bed rest had a positive effect on lean mass HMB: −1.2% ± 0.9% vs. control (CON): −4.6% ± 1.4%) and knee extensor strength in older volunteers [30]. These data were broadly consistent with the protective effect observed following leucine supplementation during 14 days of bed rest in middle-aged adults.

## 4. Translating Acute Research Studies: Concept to Practice?

While data from acute metabolic studies may be compelling, logical and reproducible, their value often lies in the establishment or support of a concept or theory and not a specific or immediately translatable recommendation. For example, in a series of acute studies, our laboratory and others have highlighted the potential benefits of evenly distributing protein intake across three daily meals, instead of the more common practice of skewing protein and energy intake towards the evening meal [29,31]. Specifically, we demonstrated that in healthy, ambulatory, young adults, an even distribution of protein (30 g breakfast, 30 g lunch, 30 g dinner) promoted a 25% greater 24 h muscle protein synthetic response than the same total amount of protein consumed in a skewed pattern (10 g breakfast, 15 g lunch, 65 g dinner) [31]. While consuming approximately 30 g protein/meal may be challenging or impractical for some and insufficient for others [13,15,32], the general “even protein distribution concept” may have the potential to benefit individuals exposed to the anabolic resistance and catabolic stress of disuse [24,31]. If the benefits of the protein distribution concept can be demonstrated in longer duration trials and outcome studies, it may represent an incremental positive step. However, in a generally healthy population, the magnitude and rapidity of potential benefits associated with simply moving from a “heavily skewed” to “evenly distributed” protein intake should not be overestimated. For example, in a free-living aging population, sarcopenia accounts for approximately 0.8%–1.0% loss of lean body mass each year. Over a three-month period, a common duration for many nutrition intervention studies, a 70 kg older adult could expect to lose ~175 g of lean mass. Even if the habitual consumption of a skewed protein diet were wholly responsible for this loss, would it be reasonable to expect a meaningful improvement associated with simply moving to an “evenly distributed” protein intake? Although dual energy X-ray absorptiometry (DXA) is typically used to determine body composition, small-to-modest changes in lean body mass (e.g., ±250 g) may not reliably meet the detection threshold for current whole body measures [33,34]. Researchers, clinicians and the media need to be more aware of the conceptual differences and realistic outcomes associated with nutrition-based strategies designed to preserve muscle mass/function over the long term vs. strategies designed to build muscle or counter accelerated catabolism (Figure 1).

For individuals experiencing difficulties consuming a sufficient quantity or quality of protein at each meal, amino acid or protein supplementation may be a beneficial option. Whey protein is generally considered to be the “gold standard” protein supplement. It is has a favorable essential amino acids profile and is widely used in research studies and clinical and athletic environments [15,35,36,37]. More recently, researchers have noted that leucine or the leucine metabolite, HMB, supplementation (3–4 g/meal) can provide an “anabolic boost” to smaller protein meals (10–15 g protein), through their ability to directly and indirectly activate the mammalian target of rapamycin complex 1 (mTORC1) signaling pathway, the major molecular activator of muscle protein synthesis in the cell. The comparatively smaller pathway serving size (3–4 g/meal) of leucine and HMB may therefore be more easily tolerated than traditional higher-volume supplements (Table 2) [26,30,38]. For individuals with reduced energy requirements, moderating the quantity of protein and leucine consumed at each meal could also help control total energy intake and ensure other micro-and macronutrient needs are met.

While most acute metabolic studies examining leucine’s effect of skeletal muscle are positive [7,26,27,38,43], the potential of longer-term supplementation to influence phenotypic or functional outcomes is less certain. For example, while a sustained increase in skeletal muscle protein synthesis can be achieved by adding leucine to meals (4 g leucine/meal, three meals) over a two-week period [26], there may also be concomitant time-course or saturation effect that needs to be considered [44,45]. In a recent 14 day bed rest study in middle-aged adults, leucine supplementation (0.06 g/kg/meal) had a robust protective effect on lean body mass during the initial seven-day of inactivity. However, during the final days of bed rest, the rate of loss in leucine-supplemented subjects was the same as the control condition [7]. Clearly, there are many potential areas of disconnect between acute and longer-term nutrition trials that extend beyond the inherent heterogeneity and variability in study populations.

## 5. Cellular Mechanisms of Inactivity-Induced Muscle Loss

Protein kinase B (Akt/PKB) is an important molecular switch that positively regulates skeletal muscle mass [46,47,48] in response to exercise and nutrition by enhancing protein synthesis and inhibiting protein degradation pathways. mTORC1 is the primary downstream target of Akt that modulates muscle protein synthesis. mTORC1 is a serine/threonine kinase responsible for initiating protein translation by directly phosphorylating its downstream targets p70-S6K (RPS6KB1) and 4E-BP1 (EIF4EBP1) [49,50]. Leucine stimulates insulin release and indirectly activate the Akt pathway, while also acting at the mTORC1 level through an insulin independent pathway that may involve the Ras-related GTPase (Rag), Vps34 or MAP4K3. These direct and indirect mechanisms converge to activate mTOR signaling and increase protein synthesis in the muscle [43]. Akt also inhibits muscle proteolysis by repressing the action of the Forkhead box FOXO proteins and their targets, the muscle specific E3-ubiquitin ligases Muscle Atrophy F-Box (MAFbx-atrogin) and muscle Really Interesting New Gene (RING)-finger protein 1 (MuRF1) [51,52]. As a result, changes in gene and protein levels of the members of the Akt signaling pathways often parallel functional outcomes in response to anabolic and catabolic stimulation. While these markers only provide a temporal snapshot of chronic physiological processes, they are often used to decipher more complex metabolic interactions such as the combination of an anabolic and a catabolic stimulus.

Muscle disuse typically induces anabolic resistance and blunts the protein synthesis response to acute amino acid ingestion. Postprandial muscle protein synthesis decreases 45%–50% following 14 days of bed rest in older adults [53] or five days of full leg cast immobilization in young adults [54]. This relative attenuation in muscle protein synthesis rate is often paralleled by changes in the activation pattern of the members of the Akt signaling pathway. However, snapshot measures of signaling are not always consistent with expected translational outcomes and may be an artifact of the study design or time course of sampling rather than a physiological phenomenon. For example, in response to a standardized essential amino acid meal following seven days of bed rest, older adults experienced decreases in: (i) muscle protein synthesis; (ii) mTORC1 signaling (i.e., decreased phosphorylation of protein kinase B (Akt), mTOR, ribosomal protein S6 kinase 1 [50], ribosomal protein S6 (rpS6)); and (iii) amino acid transporters (reduced protein content of L-type amino acid transporter 1 (LAT1) and sodium-coupled neutral amino acid transporter (SNAT2) [20]. However, others have reported no changes in phosphorylation levels of mTOR and its downstream signaling proteins following five days of immobilization [54].

Muscle protein breakdown may also contribute to the negative effects of disuse [55]. Unfortunately, obtaining definitive results is hampered by methodological constraints. Specifically, this includes difficulties modeling muscle protein breakdown in the non-steady state (e.g., in association with meals or exercise) and the limitation associated with obtaining blood or muscle samples during a narrow assessment window (3–5 h). In one of the few studies where synthesis and breakdown were assessed concurrently, volunteers were subjected to 21 days of bed rest with a six head-down-tilt [22]. Mixed muscle protein synthesis decreased by 48.5%, while muscle protein breakdown remained unchanged. Markers of muscle protein breakdown calpain 1, calpain 3, calpastatin, MuRF1, and MuRF2 are also unresponsive to bed rest [56]. However, others have reported increases in transcript abundance of atrophic initiators in response to limb immobilization/suspension [54,57] and bed rest [10], especially following the first 2–4 days of disuse [58]. These data suggested that an acute activation of the protein degradation pathways may be the earliest contributor to disuse-induced muscle atrophy.

## 6. Inactivity and Glucose Regulation

Extended periods of reduced activity can contribute to glucose dysregulation and insulin resistance in young and older adults [11,59,60,61]. In healthy volunteers, insulin action is impaired during inactivity despite rigorous control of energy intake [2,57]. While limb immobilization/suspension induces localized insulin resistance [54], the more generalized nature of bed rest is associated with greater peripheral and whole body insulin resistance [55,56]. Some data suggest that inactivity-induced insulin resistance is primarily linked to impaired peripheral insulin action (i.e., in the muscle) rather than an inability to shut down endogenous glucose production [62].

Overweight, older volunteers subjected to 10 days of bed rest experienced a 15% decline in insulin-stimulated glucose disposal (ISGD) and an increase in fasting glucose [60]. Similarly, young men who underwent nine days of bed rest displayed a 25% decrease in ISGD [59,63]. Although the young cohort experienced a greater absolute decrement in ISGD, the post-bed rest rate of disposal of the younger and older adults was comparable (~10 mg/kg FFM/min). These observations parallel findings that younger participants experienced a greater absolute decline in postprandial insulin sensitivity in response to a mixed meal than older participants following 14 days of bed rest, but the post-bed rest values were similar [11]. While these data describe the contribution of muscle to inactivity-induced insulin resistance, the underlying mechanisms driving the changes in insulin and glucose have only been partly uncovered. In a cohort of young men, seven days of bed rest decreased (i) GLUT4 abundance (glucose transport capacity); (ii) hexokinase II levels (glucose phosphorylation); and (iii) the propagation of insulin signaling to phosphorylate glycogen synthase (reduced glucose storage) [61]. Nine days of bed rest in young men decreased expression of several transcripts in muscle associated with the oxygen phosphorylation pathway, including PGC1α [63]. The decrease in expression of genes associated with oxidative capacity indicates that the health of skeletal muscle mitochondria may be compromised during bed rest and contribute to inactivity-induced insulin resistance [64,65]. It is unknown if the reduction in skeletal muscle glucose transport and storage and oxidative capacity following bed rest that contribute to insulin resistance observed in young adults is the same mechanism that occurs in older adults.

Acute periods of low or reduced physical activity levels also disrupt insulin action. Young, non-exercising men asked to reduce step count to ~1500/day displayed peripheral insulin resistance following two weeks of reduced activity [66]. Insulin action (as measured by rate of glucose disappearance) was reduced by 39% in healthy young adults following a single extended bout (~16 h) of sitting [67]. These data highlight the rapidity and easily inducible nature of inactivity- induced insulin resistance.

In individuals with preexisting comorbidities, it is difficult to determine the extent to which intervals of inactivity due to illness or hospitalization exacerbate insulin resistance. However, in clinical populations, poorly controlled blood glucose is associated with an increase in mortality and length of stay, even if there is no history of diabetes [68,69,70]. Aggressive pharmacological interventions are often implemented to control blood glucose in older adults, increasing the risk of hypoglycemic events [70,71]. It is highly desirable to develop nutritional strategies that complement the pharmacological care needed to modulate glucose control in clinical populations.

Future studies will be useful in understanding any advantages to leveraging protein, and, in particular, leucine, to maintain the relationship between muscle health and insulin signaling during disuse. As noted earlier, leucine supplementation, may partially protect muscle mass during brief periods of inactivity [7]. Given the fact that skeletal muscle accounts for approximately 75% of insulin-mediated glucose uptake [72], protecting muscle mass during inactivity using leucine or branch-chained amino acids may offer some beneficial effect on glucose regulation [73]. In addition, replacing the traditional high-carbohydrate breakfast with a moderate amount of protein through the adoption of an evenly distributed approach to protein consumption may also help regulate insulin by reducing large glucose excursions [74].

## 7. Conclusions

Habitual dietary protein consumption practices can play an important role in muscle metabolism. However, simply increasing dietary intake to preserve muscle mass may not be the most effective or suitable approach. The primary concern associated with bluntly increasing protein intake is exceeding energy requirements via a concomitant increase in carbohydrate and fat consumption. Targeted lower-volume supplementation may be a more effective alternative than simply increasing protein intake in some populations. Leucine supplementation has been shown to protect skeletal muscle by mitigating the loss of muscle mass, strength, and endurance associated with disuse. Leucine is a promising candidate to counter the negative effects of disuse in clinical environments; however, more research is needed to link the mechanisms of action with clinically relevant translational/functional outcomes. Moving forward, we must continue to critique and challenge our understanding of how protein intake can be optimized to combat the negative effects of disuse on body composition and muscle health for various populations and in different circumstances.

## Figures and Tables

**Table 1:** The negative consequences of disuse parallel changes observed in clinical populations and sarcopenic or frail older adults.

Decline in basal energy expenditure
Reduced insulin sensitivity
Reduced muscle strength
Reduced physical performance
Increased risk for falls
Increased health-related expenses
Increased morbidity
Increased mortality

**Table 2:** Quantity of supplemental protein (powdered form) required to provide 3 g of leucine. Nutrition information from the USDA National Nutrient Database or peer-reviewed publications. Estimates of protein content for micellar casein and whey hydrolysate are based on 90% protein concentration.

Protein Supplement	Product (g)	Protein (g)	Leucine (g)	Energy (kcal)
Collagen Hydrolysate/Gelatin [39,40]	122.2	104.6	3	409
Pea Isolate [41]	46.9	27.8	3	182
Soy Isolate [39]	44.2	39.0	3	148
Micellar Casein [42]	36.5	32.9	3	167
Whey Hydrolysate [42]	27.9	25.1	3	130
Leucine	3	3	3	12

## Abbreviations

The following abbreviations are used in this manuscript:

4E-BP1	Eukaryotic Translation Initiation Factor 4E-Binding Protein 1
Akt/PKB	Protein Kinase B
DXA	Dual Energy X-ray Absorptiometry
EAR	Estimated Average Requirement
FOXO	Forkhead box
GLUT4	Glucose Transporter 4
HMB	Beta-Hydroxy-beta-Methylbutyrate
ISGD	Insulin-Stimulated Glucose Disposal
IOM	Institute of Medicine
LAT1	l-type Amino Acid Transporter 1
MAFbx	Muscle Atrophy F-Box
MAP4K3	Mitogen-Activated Protein Kinase Kinase Kinase Kinase 3
miRNA	micro RNA
mTORC1	Mammalian Target of Rapamycin Complex 1
MuRF	Muscle Really Interesting New Gene (RING)-Finger
p70-S6K	Ribosomal Protein S6 Kinase beta-1
RDA	Recommended Dietary Allowance
rpS6	Ribosomal Protein S6
SNAT2	Sodium-coupled Neutral Amino Acid Transporter
Vps34	Vacuolar Protein Sorting 34
